# Views on increased federal access to state and local National Syndromic Surveillance Program data: a nominal group technique study with state and local epidemiologists

**DOI:** 10.1186/s12889-023-15161-5

**Published:** 2023-03-06

**Authors:** Cason D. Schmit, Brooke Willis, Hayleigh McCall, Alyaa Altabbaa, David Washburn

**Affiliations:** 1 Program in Health Law and Policy, Texas A&M University School of Public Health, 212 Adriance Lab Rd, TX 77843 College Station, USA; 2 Texas A&M University School of Law, 1515 Commerce St, 76102 Fort Worth, TX USA; 3 Council of State and Territorial Epidemiologists, 2635 Century Parkway NE, Suite 700, Atlanta, GA 30345 USA; 4 Public Health Informatics Institute, 325 Swanton Way, Atlanta, GA 30030 USA; 5 InductiveHealth Informatics LLC, 3107 Clairmont Road N NE, Suite C, Atlanta, GA 30329 USA

**Keywords:** Public health surveillance, Syndromic surveillance, data dissemination, Privacy, Confidentiality, Policy

## Abstract

**Background:**

US public health authorities use syndromic surveillance to monitor and detect public health threats, conditions, and trends in near real-time. Nearly all US jurisdictions that conduct syndromic surveillance send their data to the National Syndromic Surveillance Program (NSSP), operated by the US. Centers for Disease Control and Prevention. However, current data sharing agreements limit federal access to state and local NSSP data to only multi-state regional aggregations. This limitation was a significant challenge for the national response to COVID-19. This study seeks to understand state and local epidemiologists’ views on increased federal access to state NSSP data and identify policy opportunities for public health data modernization.

**Methods:**

In September 2021, we used a virtual, modified nominal group technique with twenty regionally diverse epidemiologists in leadership positions and three individuals representing national public health organizations. Participants individually generated ideas on benefits, concerns, and policy opportunities relating to increased federal access to state and local NSSP data. In small groups, participants clarified and grouped the ideas into broader themes with the assistance of the research team. An web-based survey was used to evaluate and rank the themes using five-point Likert importance questions, top-3 ranking questions, and open-ended response questions.

**Results:**

Participants identified five benefit themes for increased federal access to jurisdictional NSSP data, with the most important being improved cross-jurisdiction collaboration (mean Likert = 4.53) and surveillance practice (4.07). Participants identified nine concern themes, with the most important concerns being federal actors using jurisdictional data without notice (4.60) and misinterpretation of data (4.53). Participants identified eleven policy opportunities, with the most important being involving state and local partners in analysis (4.93) and developing communication protocols (4.53).

**Conclusion:**

These findings identify barriers and opportunities to federal-state-local collaboration critical to current data modernization efforts. Syndromic surveillance considerations warrant data-sharing caution. However, identified policy opportunities share congruence with existing legal agreements, suggesting that syndromic partners are closer to agreement than they might realize. Moreover, several policy opportunities (i.e., including state and local partners in data analysis and developing communication protocols) received consensus support and provide a promising path forward.

**Supplementary Information:**

The online version contains supplementary material available at 10.1186/s12889-023-15161-5.

## Background

Public health data are critical to the success of public health organizations’ mission to promote community health [1]. Syndromic surveillance has been used to detect and monitor community health issues and trends and better understand the health impact of acute events [2]. Syndromic surveillance applications are varied and broad, including monitoring salmonella [3], influenza-like illnesses [3], hazardous material exposures, natural disasters [4], violence [5], mental health [6], climate-related illness [7], Tick-borne illness [8], and opioid overdoses [9]. Forty-nine states participate in the National Syndromic Surveillance Program (NSSP), including 71% of U.S. emergency departments.[10].

Syndromic surveillance can incorporate diverse data sources, including vital statistics, environmental, and laboratory data. However, one of the primary syndromic surveillance data sources—and our focus—consists of a subset of electronic health record data from emergency departments and urgent care centers that are shared with public health authorities in near-real time. Epidemiologists use validated definitions to classify electronic health record data fields—like chief complaints and diagnosis codes—into syndromes that can be tracked and monitored. This process is less exact than other surveillance methods—like case reporting—but near real-time data make syndromic surveillance a valuable support tool for public health agencies to better understand emerging and ongoing public health events.

During the COVID-19 pandemic, syndromic surveillance has been a valuable tool for state and local public health agencies to monitor, in near real-time, COVID-19-associated trends in their jurisdictions [11]. However, current data sharing policies between state, local, and federal partners were an obstacle to federal efforts to utilize syndromic surveillance to monitor COVID-19. This work is a direct response to those challenges.

Many challenges are derivative of U.S. federalism and the distinct public health roles of state, local, territorial, and federal governments. The substantial police powers reserved to states through the 10th Amendment of the U.S. Constitution give states the primary public health responsibility within their jurisdictions. The federal government serves an essential supporting role, providing resources, assistance, and monitoring interjurisdictional public health issues and threats. These distinct jurisdictional roles, compounded by associated relationship and political dynamics, have created data sharing challenges. Within the U.S. public health surveillance system, these roles, relationships, and political dynamics manifest in the data use agreements (DUAs) between state, local, territorial, and federal public health partners [12].

**Fig. 1:**
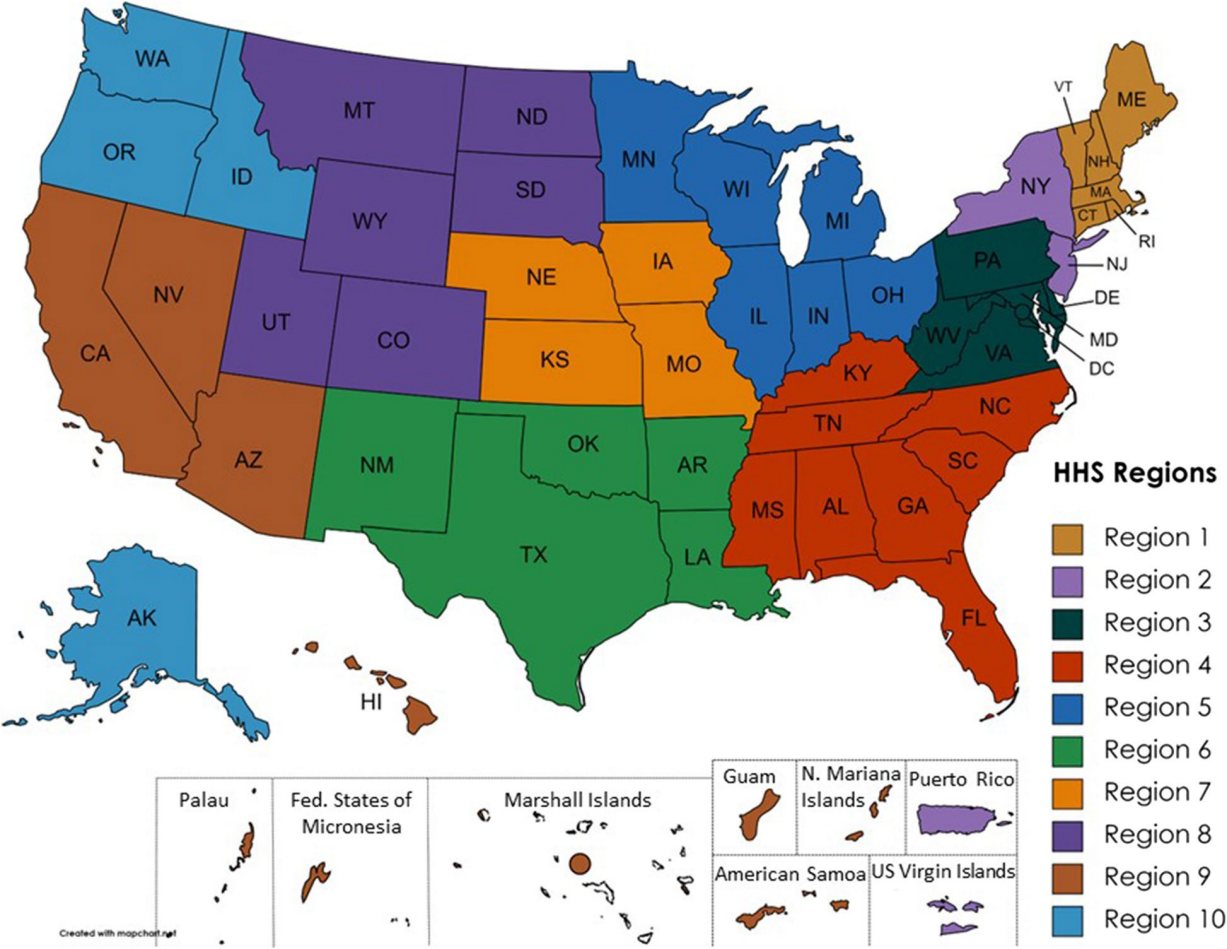
Federal access to the National Syndromic Surveillance Program is limited to Department of Health and Human Services regions

Currently, most jurisdictions send their syndromic surveillance data to NSSP, which operates on servers maintained by the Centers for Disease Control and Prevention (CDC). However, DUAs severely restrict federal use of state and local data within NSSP by limiting the ability of the federal government to access state or local NSSP data below the U.S. Department of Health and Human Services (HHS) region level [13] (Fig. 1). Consequently, federal NSSP personnel have no way to determine, for example, whether an observed increase of a syndrome in HHS Region 10 is (1) an isolated event in Washington, (2) unrelated, but similar, events in Washington and Alaska, or (3) related events in Washington and Oregon [14]. Currently, NSSP DUA permissions require federal NSSP personnel to request expanded access separately from all jurisdictions within the HHS region to distinguish these scenarios. Consequently, the existing NSSP federal access policy could be fairly described as a policy that could result in non-detection of interjurisdictional events.

If a detected public health event is significant, the transaction cost on the federal government to separately negotiate increased access with every jurisdiction—each under strain from their separate public health responses—would be inordinate. Therefore, the current policy framework is a substantial challenge to the federal use of NSSP data to respond to events—like bioterrorism and epidemic disease—that impelled initial federal support for NSSP [15, 16].

Syndromic surveillance technology, specifically the NSSP BioSense Platform, has evolved since its introduction in 2002 with its redesign into BioSense 2.0 in 2010, and final evolution into NSSP in 2014 [17]. Throughout each evolutionary iteration, NSSP BioSense has identified and addressed new challenges, using policy and technology to balance diverse stakeholders’ interests [17]. For example, early attitudes of public health professionals towards the CDC and adoption of BioSense were negatively affected by the CDC essentially bypassing state and local public health departments by eliciting data directly from hospitals, and instances of federal misinterpretation of early state and local syndromic surveillance data when analytical standards and processes norms were still nascent [4, 17]. These sentiments influenced the policy and technical decisions of the initial BioSense redesign, BioSense 2.0, including giving states greater control and independence. However, the BioSense 2.0 changes made it difficult for CDC to offer quality assurance and and technical assistance, challenges that were addressed in the creation of NSSP [17]. While several incentive programs have increased syndromic surveillance adoption [18], there are several remaining concerns that public health officials and stakeholders have with increased federal access to and use of NSSP data [19]. Given the CARES Act’s (2020) $500 million data modernization initiative, a new evolutionary step for NSSP seems likely, if not imminent. Examining the existing challenges and barriers faced by NSSP and its federal, state, and local users is critical to crafting an effective data modernization strategy.

The existing access policies were a significant challenge and inhibitor to the initial federal response to COVID-19 [16]. While many jurisdictions granted increased federal access to their NSSP data, other jurisdictions did not. Given the public health emergency in early 2020, the White House COVID-19 Task Force requested and received access to state and local COVID-19 syndromic surveillance data from CDC NSSP [20]. Importantly, the increased federal access to state and local NSSP data was an emergency action taken by the federal government and did not follow the jurisdiction-controlled data access provisions described in the NSSP DUA. These emergency actions created additional strain on state/local-federal relationships.

It should be noted that the federal access restrictions for state and local public health data are anomalous compared to other national public health surveillance systems. While NSSP restricts federal access to data aggregated at the HHS-region level, the National Notifiable Disease Surveillance System, the Behavioral Risk Factor Surveillance System, National Vital Statistics System, and the Overdose Data to Action surveillance systems all permit federal access to more granular jurisdictional data [21–24].

There are numerous policy approaches that could be used to these data sharing challenges. First, the federal government could mandate increased data sharing; however, this could be considered heavy-handed and would likely neglect some of the state concerns that impelled the imposition of these restrictions (e.g., data misinterpretation, bypassing state and local health officials) [17, 25]. Second, the federal government could incentivize greater data sharing by conditioning federal funding on greater access to NSSP data; but this too could damage federal relationships with their state and local partners and push jurisdictions with their own syndromic surveillance systems out of the national system. Finally, the federal government could identify a solution via an intergovernmental agreement, such as a DUA. This latter option has emerged as the preferred approach to govern data sharing between federal, state, and local partners [26].

This research represents the first step to identify policy opportunities to address the challenges sharing state and local NSSP data with federal public health partners. The Council of State and Territorial Epidemiologists (CSTE), in collaboration with the CDC and Texas A&M University, convened a workgroup comprised of state and local epidemiologists and leaders to develop considerations and implementation strategies for revising federal NSSP data access policies. This article describes the perceived benefits and concerns regarding increased federal access to NSSP data reported by leadership within local, state, and national public health organizations.

### Methodology

The purpose of this workgroup was to identify perceptions specifically pertaining to NSSP for the purpose of identifying strategies to revise NSSP policy. As a result, this work was determined not to be human subjects research by the Texas A&M University Institutional Review Board (IRB2021-0932).

Potential workgroup members were identified and requested to participate by CSTE based on their decision-making position at the health department, surveillance/informatics expertise, geographical region, and engagement in surveillance policy discussions. Many of these individuals were active participants and contributors within the NSSP Community of Practice or through engagement in CSTE activities. Twenty-five representatives of state and local health departments were invited individually via email to participate or designate an appropriate surrogate for the workgroup. In addition, three individuals representing national public health organizations (CSTE, Association of State and Territorial Health Officials [ASTHO]) participated in the discussion.

The workgroup meeting followed a Nominal Group Technique (“NGT”) approach. The NGT method facilitates individual idea generation by promoting equal input in group settings, dampening the effects of dominant members, and enabling idea prioritization [27–30]. For example, the NGT approach utilizes individual idea generation and non-confrontational idea clarification in its beginning stages to ensure that contributions from all members are included and considered, and the prioritization of ideas in the latter stages is done confidentially. The NGT method is among the most used qualitative methods for group decision-making processes to identify and clarify problems [27–29].

Three questions—developed in consultation with CDC and CSTE representatives—were asked during the NGT workgroup:

1. In what ways can increased federal access to state syndromic surveillance data (at the state or local level) benefit or support state public health activities?;
2. What concerns you about increasing federal access to state syndromic surveillance data at the state or local level?; and.
3. What rules, restrictions, guidelines, or codes of conduct could be implemented in the NSSP DUA or CDC policies that might address a concern addressed by you or a fellow workgroup member?

The NGT workgroup was conducted on September 2, 2021, via an online platform (Zoom) to allow nationwide participation during the COVID-19 pandemic. During the first 45 minutes, we provided background information on the issues surrounding federal access, and the impetus, purpose, and scope of the workgroup, assisted by three federal CDC NSSP employees, who answered participant questions. Afterward, all federal personnel left the meeting to avoid influencing the NGT process.

Participants were given 15 minutes to independently generate ideas relating to questions 1 (benefits) and 2 (concerns). Then participants were divided into five breakout rooms for 20 minutes to clarify their ideas for each question in a round-robin format, recording ideas on a web-based collaborative worksheet (Google Doc). Participants were then given eight minutes to individually generate ideas for question 3 (policies) followed by a 10-minute small group session to clarify and record those ideas. Finally, participants were divided into three larger groups—each assigned one research question—and tasked with grouping all the ideas into themes with assistance from the research team. For example, the research team reviewed the themes to ensure that all the independently-generated ideas were represented in themes and made minor edits for clarity.

We used a web-based survey software (Qualtrics XM) to facilitate theme prioritization. Participants evaluated the themes using a 5-point Likert scale (i.e., very important, important, moderately important, slightly important, not important) and ranked their top-3 most important themes for each question. We calculated an aggregate ranking score by assigning 3, 2, and 1 points to participants top 1, 2, and 3 ranked themes, respectively, and summing values for all participants. Open-ended questions permitted participants to share additional thoughts. We opened the web-based survey to a limited number of other identified workgroup member candidates—also state and local health department officials—who did not make the workgroup call for various reasons (e.g., scheduling, pandemic response).

## Results

### Participant characteristics

The workgroup call had 21 participants, 3 worked for local health departments, 2 worked for CSTE, 1 worked for ASTHO, and the remaining 15 worked for state health departments. There was broad regional representation among participants.

The small groups ranged from 2 to 5 participants. With the exception of one group of 2—who had difficulty joining a Zoom “breakout room”—the small groups were within the optimal size for NGT studies [29]. Fifteen individuals—all representing state or local health departments—completed the theme prioritization survey. Of those, only 3 individuals did not participate in the workgroup. Several participants did not complete the entire NGT exercise due to work commitments (e.g., pandemic response).

### Benefits of increased federal access to state or local NSSP data

The workgroup generated ideas that fit into five benefit themes. All those themes are presented in Table 1. See the supplemental materials for example ideas for each theme. Of the benefit themes, the most important were improved cross-jurisdiction collaboration efforts, followed by improved syndromic surveillance practice, scoring highly in the Likert and ranking questions. Average Likert importance scores indicated that all benefit themes were at least “moderately important,” and 2 of the 5 themes had average Likert scores indicating that they were at least “important.”

**Table 1:** State and local epidemiologists’ evaluation of benefits of increased federal access to NSSP data, 2021

Identified Benefit^a^	Mean importance Likert score^b^	Rank 1 (n)	Rank 2 (n)	Rank 3 (n)	Aggregate rank score^c^
Improved cross-jurisdiction collaboration efforts	**4.53**	6	3	3	**27**
Improved syndromic surveillance practice	**4.07**	2	7	3	**23**
Technical assistance + expertise	**3.53**	4	2	1	**17**
Enhanced state capacity	**3.73**	2	1	5	**13**
Enhanced federal surveillance capacity (e.g., providing national pictures, completing data requests normally handled by states, increased cross-jurisdictional awareness)	**3.40**	1	2	3	**10**

Themes were generated in response to the question: “In what ways can increased federal access to state syndromic surveillance data at the state or local level benefit or support state public health activities?”

^a^Example ideas for each theme can be found in the Supplemental materials

^b^In calculating the mean Likert scores, options were scored 1–5 with Very Important = 5, Important = 4, Moderately Important = 3, Slightly Important = 2, Not Important = 1

^c^In calculating the rank score, items ranked 1, 2, and 3 were assigned scores of 3, 2, and 1 respectively. The aggregate rank score is the sum of all respondents’ ranking scores

Open-ended responses suggested possible divergent opinions on increased federal access. One participant felt that “[t]he benefits are very theoretical at this time and have huge down sides if not managed properly and with appropriate accountability at CDC for people who misuse the system.” However, another participant stated, “I think federal access to the NSSP data will increase awareness and better understanding of how to use the data, limitations and caveats, and furthermore collaboration for utilizing the tools in the BioSense Platform.”

### Concerns about increased federal access to state or local NSSP data

The workgroup generated ideas that fit within nine concern themes. All those themes are presented in Table 2. See the supplemental materials for example ideas for each theme. The two concerns with the highest Likert importance scores and ranking scores were the federal government independently sharing data or initiating public health action without notifying states, followed closely by misinterpretation of data. Average Likert importance scores indicated that all concern themes were at least “moderately important,” and 6 of the 9 themes had average Likert scores indicating that they were at least “important.”

**Table 2:** State and local epidemiologists’ evaluation of concerns of increased federal access to NSSP data, 2021

Identified Concern^a^	Mean importance Likert score^b^	Rank 1 (n)	Rank 2 (n)	Rank 3 (n)	Aggregate rank score^c^
Federal government independently sharing data or initiating public health action without notifying states.	**4.60**	5	4	1	**24**
Misinterpretation of data	**4.53**	5	3	0	**21**
Privacy and confidentiality concerns, including data sensitivity, restriction of certain fields, and public perception of increased data sharing.	**4.47**	4	1	1	**15**
Freedom of Information Act (FOIA) Issues	**4.40**	0	4	2	**10**
Adequacy of and adherence to data sharing rules (including agreements codes of conduct, etc.)	**4.53**	0	1	5	**7**
Inadequate, excessive, or inappropriate communication regarding data uses	**4.33**	1	0	2	**5**
Publishing the data can decrease jurisdictional credibility	**3.27**	0	1	2	**4**
Increasing the burden on jurisdictions	**3.47**	0	0	2	**2**
Negative effect on collaborations leading to presentations or publications	**3.33**	0	1	0	**2**

Themes were generated in response to the question: “What concerns you about increasing federal access to state syndromic surveillance data at the state or local level?”

^a^Example ideas for each theme can be found in the Supplemental materials

^b^In calculating the mean Likert scores, options were scored 1–5 with Very Important = 5, Important = 4, Moderately Important = 3, Slightly Important = 2, Not Important = 1

^c^In calculating the rank score, items ranked 1, 2, and 3 were assigned scores of 3, 2, and 1 respectively. The aggregate rank score is the sum of all respondents’ ranking scores

Several open-ended responses were instructive to interpreting these concerns. One participant stated, “all concerns listed are of great importance and each outweigh the benefits of increased federal access without restrictions.” Others elaborated on the potential to burden states, with one saying:

As with past experiences during COVID, when CDC releases data at state level that states are also releasing and analyzing, any discrepancies can cause unnecessary public alarm and leads to extensive time at the state and local level evaluating and explaining.

Another expressed concern that there will be “only negative impacts from [the federal government] overburdening states with data interpretation questions.” One participant stressed the importance of balancing the potential benefits with the concerns.

### Policy options for increased federal access to state or local NSSP data

The workgroup generated ideas that fit within twelve policy option themes to address concerns about greater federal access to state or local NSSP data. All those themes are presented in Table 3. See the supplemental materials for example ideas for each theme. The most important themes were involving state and local partners in data analysis, followed by creating communication protocols. Average Likert importance scores indicated that all policy themes were at least “moderately important,” and 9 of the 12 themes had average Likert scores indicating that they were at least “important.” Nearly all participants (14/15) rated “involving state and local partners in data analysis” as “very important.”

**Table 3:** State and local epidemiologists’ evaluation of policy opportunities for increased federal NSSP access, 2021

Identified Rule, Restriction, Guideline or Code of Conduct^a^	Mean importance Likert score^b^	Rank 1 (n)	Rank 2 (n)	Rank 3 (n)	Aggregate rank score^c^
Involving state and local partners in data analysis	**4.93**	4	4	2	**22**
Create communication protocols between CDC and state or local governments	**4.53**	4	2	1	**17**
Make DUA applicable to all federal recipients of NSSP data	**4.53**	1	1	3	**8**
Restrict data access for specific purposes or events	**3.73**	1	2	1	**8**
Establish audit and documentation process for data access and analysis	**4.33**	0	3	1	**7**
Create standards for removing access	**4.07**	2	0	1	**7**
Restrict data access to specific users (as opposed to groups of users)	**3.53**	1	1	1	**6**
Establish restrictions on data publication	**4.13**	1	1	0	**5**
Allow optional participation in greater federal access	**4.00**	1	1	0	**5**
Include procedure for DUA renewal	**4.07**	0	0	2	**2**
Require training on code of conduct	**3.67**	0	0	2	**2**
Clarify breach responsibility	**4.07**	0	0	1	**1**

Themes were generated in response to the question: “What rules, restrictions, guidelines, or codes of conduct could be implemented in the NSSP DUA or CDC policies that might address a concern addressed by you or a fellow workgroup member?”

^a^Example ideas for each theme can be found in the Supplemental materials

^b^In calculating the mean Likert scores, options were scored 1–5 with Very Important = 5, Important = 4, Moderately Important = 3, Slightly Important = 2, Not Important = 1

^c^In calculating the rank score, items ranked 1, 2, and 3 were assigned scores of 3, 2, and 1 respectively. The aggregate rank score is the sum of all respondents’ ranking scores

Several open-ended responses provided additional details on user access policies. For example, one participant said:

I would be amenable to having a small/core group of analysts who are named and whose role/purpose is clearly delineated that have constant access to our detailed data. Other federal staff who would like access to detailed state data should require approval from sites after their purpose is clearly defined to improve collaboration with sites… I’m not in favor of NSSP granting other federal staff access to additional data for projects that come along without some sort of agreement or notification to [the health department providing data to NSSP].

Another participant recommended:

[T]he federal request for access be specific in terms of geographic level of access, and purposes. If the request is unlimited access to all data for any topic or purpose, it will be harder to support/approve. This is especially true for line level requests or county-level access.

One participant suggested the importance of accountability mechanisms, saying that without a process for removing access “there are no teeth to the required codes of conduct, DUAs, etc.”

Several participants had specific comments relating to the NSSP DUA. One participant wants to ensure that “[a]ll federal users with access should be beholden to the DUA between the site and NSSP.” Another user was concerned that a “piecemeal approach” to DUAs was insufficient:

One DUA with only on[e] program or center like NSSP, does not cover all other users [e.g., other federal agencies]... The more that can be covered under a larger agreement, reinforced by the code of conduct, protocols for maintaining access to the data, training for use of the data at multiple levels of users, the fewer the concerns can become.

Participants had other specific suggestions. One participant noted they were concerned by the “disregard for agreements in place due to pressures within federal government or emergency status” and argued that “it is important that any rules/policies/guidelines are emergency-proof so they don’t just get thrown out the window in the event of an emergency.” Another participant said they would also like to see “some sort of policy that invites review/comment of analyses that uses a site’s data prior to their release/submission and also some basic publication standards (e.g., suppression of small numbers).” Lastly, a participant recommended “there needs to be a way to have some standardization across jurisdictions for direct access to the data and how [it’s] used.”

## Discussion

As an NGT study, these results are intended begin a problem-solving process. Here, we have identified potential benefits, concerns, and policy opportunities for increased federal access to state and local syndromic surveillance data. As such, this work is an initial step to understand and enhance national syndromic surveillance and its policy framework.

Several of our findings reflect themes that have persisted through the history and evolution of NSSP [17]. For example, concerns that the CDC might bypass state and local health departments and contact health facilities were raised for BioSense 1.0 [17]. These concerns are similar to our top-rated concern “federal government independently sharing data or initiating public health action without notifying states.” Similarly, increased interjurisdictional collaboration was a priority in the development of BioSense 2.0, and that impetus is related to our top-rated benefit of increased federal access: “improved cross-jurisdiction collaboration efforts” [31, 32]. In Gould, Walker, and Yoon’s historical review of NSSP, they note how challenges and concerns impelled each evolution of BioSense, but the solutions were sometimes imperfect, leading to new challenges [17]. For example, BioSense 2.0 was supposed to facilitate intergovernmental collaborations [31, 32]. However, it accomplished this by shielding syndromic surveillance data from CDC access on servers operated by ASTHO, which prevented CDC from assisting with quality assurance and technical assistance [17]. NSSP fixed this issue by moving syndromic surveillance data back onto CDC systems, but it established a default—via the DUAs—of non-sharing between federal, state, and local partners. Our findings suggest that the US syndromic surveillance community is still searching for a policy solution that appropriately enables benefits while mitigating concerns. Moreover, our findings may provide insights in why data sharing was a challenge at the onset of the COVID-19 pandemic.

However, at first glance, the NSSP policy framework—that limits federal access to state and local NSSP data—seems contrary to World Health Organization (WHO) Ethical Guidelines on Public Health Surveillance, which state “[w]ith appropriate safeguards and justification, those responsible for public health surveillance *have an obligation to share data with other national and international public health agencies*” (emphasis added) [33, 34]. Similarly, both the American Public Health Association’s Code of Ethics and the Public Health Leadership Society’s Principles for the Ethical Practice of Public Health emphasize that date should be disseminated appropriately to enable public health actions and decision-making [35, 36]. However, our findings suggest considerations specific to syndromic surveillance data that might warrant appropriate caution.

First, our results reveal significant concern that syndromic surveillance data can be easily misinterpreted. Syndromic surveillance data emphasizes timeliness of data sharing between the facility and public health, and as a result may be messier than other public health data (e.g., case reports). Moreover, electronic health record data elements used in syndromic surveillance are not standardized nationally, making them susceptible to local variations in data entry (e.g., differences in coding or chief complaint documentation) or submission conventions. Benign local events, like festivals, could produce an anomalous result in syndromic surveillance queries, sparking alarm in analysts lacking local knowledge. With this misinterpretation risk, the common benefit—a critical public health ethics principle—of greater syndromic surveillance data sharing is less certain.

Second, despite all the benefits of increased federal access to NSSP data scoring highly, open-ended responses suggest some skepticism of the public health justification for greater federal data sharing. Clarifying the public health rationale for greater syndromic surveillance data sharing is essential because the ethical obligation to share public health surveillance data assumes the existence of an appropriate justification [33].

Third, we identified some concerns about the sufficiency of existing safeguards to support greater federal data sharing. The WHO guidelines positing an obligation to share public health surveillance data assumes the existence of “appropriate” safeguards (i.e., good governance) [33]. Here, many highly prioritized concerns and policy options—including access restrictions, adequacy of data sharing rules, audits, training, confidentiality, and DUA issues—implicitly question the sufficiency of existing safeguards.

Yet, our results provide good reasons to be skeptical that the current policy limiting federal access is intractable in the current push for data modernization. We found overwhelming support for involving state and local partners in federal data analyses, which could substantially mitigate data misinterpretation risks. Better federal communication of the public health justification and anticipated benefits of increased federal access can promote critical syndromic surveillance partner support. Additional safeguards—such as the ones identified here—can be implemented to support ethical public health data sharing. Moreover, past data sharing precedents for other surveillance systems can serve as models for greater data sharing in syndromic surveillance [37].

Interestingly and surprisingly, the existing NSSP DUAs at least partially address all but three of the policy opportunities identified by this workgroup (i.e., audit process, access restriction standards, breach responsibility) [13, 34, 38]. This raises three non-exclusive possibilities. First, existing policies could be insufficient for state and local partners to support greater federal NSSP access. Second, state and local partners might not fully recognize the existing protections. Third, other factors—such as relationship and political factors—might obfuscate and complicate data sharing issues between federal, state, and local governments. Regardless, the substantial congruence between prioritized policy opportunities and the existing legal agreements suggests that federal, state, and local partners are closer to agreement than they might realize [34].

Critically, the overwhelming barrier to greater U.S. syndromic surveillance data sharing are existing DUA policies [34]. While CDC NSSP is technically capable of accessing state and local data within NSSP, DUAs between state/local governments and CDC NSSP expressly restrict access to syndromic data aggregated below the HHS-region level. These policies are reinforced by federal, state, and local partners’ political and relationship dynamics [26]. For example, the feedback suggested that federal emergency actions to access state syndromic surveillance data in response to COVID-19 strained state/local-federal relationships (e.g., beliefs that agreements were “thrown out the window”). These barriers will likely challenge future public health data sharing and data modernization initiatives.

We believe this study is the first to assess and document state and local epidemiologists’ views on the benefits, concerns, and policy opportunities related to increased federal access to state NSSP data. Our findings are crucial in a time of pandemics and during the push for public health data modernization. Moreover, workgroup feedback suggests that the NGT successfully engaged feedback from both loud and quiet voices within the syndromic surveillance community. After seeing preliminary results, one workgroup member said, “It’s just nice to see in a [results] table where folks may not feel as comfortable sharing those thoughts as openly. But seeing this here, it’s helpful having it documented.”

Importantly, this work is the first part of a larger investigation of issues surrounding federal access to state and local NSSP data. Planned activities include key informant interviews, additional workgroup sessions, evaluations of policy options, and qualitative analyses. These activities will build on these findings and explore nuance within the ideas identified by the NGT participants.

### Limitations

This work is predominantly qualitative, so care should be exercised when interpreting our findings. Workgroup members were selected based on desired criteria (i.e., geographic representation, activity in syndromic surveillance community, decision-making authority, expertise); consequently, selection bias is probable. Accordingly, our findings cannot be considered representative of all state or local epidemiologists. Similarly, perspectives from federal or national public health organizations were not represented or under-represented in our results, which might have biased or limited some feedback toward state and local perspectives. For example, benefits or risks that accrue at the national level could be underrepresented in our results. Additionally, this work was completed during a pandemic with participants having substantial responsibilities informing governmental responses. We expect that these responsibilities were a significant reason why some participants could not complete the final ranking exercise.

## Conclusion

Public health data modernization is imminent in the U.S. Impelled by the lessons of the COVID-19 pandemic, recent and pending federal actions push for improvements in public health data systems [15, 39, 40]. In a world where pandemics and public health crises ignore political boundaries, any public health data modernization initiative must address the challenges posed by U.S. federalism and the data sharing relationships between the federal. state, and local governments [41, 42]. This study describes the critical perceptions of state and local partners on sharing syndromic surveillance data with the federal government.

Critically, trust and relationships are crucial to data sharing. Our findings capture essential insights into the trust and relationship that exist between federal, state, and local public health partners. While these results identify important challenges, they also identify common ground and promising opportunities to improve public health data systems. Consequently, these findings may have significance to data modernization efforts for other public health data systems beyond syndromic surveillance.

## Supplementary Information

**Additional file 1.**

## Data Availability

The datasets generated and/or analyzed during the current study are not publicly available to protect the privacy of the public figures who participated but are available from the corresponding author on reasonable request.

## Abbreviations

ASTHO: Association of State and Territorial Health Officials
CDC: Centers for Disease Control and Prevention
CSTE: Council of State and Territorial Epidemiologists.
DUA: Data use agreement
HHS: U.S. Department of Health and Human Services
NGT: Nominal Group Technique
NSSP: National Syndromic Surveillance Program
WHO: World Health Organization
