# Supplementary material for: Views on increased federal access to state and local National Syndromic Surveillance Program data: a nominal group technique study with state and local epidemiologists
Source: BMC Public Health. 2023 Mar 6;23:431. doi: 10.1186/s12889-023-15161-5 (PMC9987142; doi:10.1186/s12889-023-15161-5)
Supplement: Supplementary file 1 — Additional file 1. [file 12889_2023_15161_MOESM1_ESM.pdf]

SUPPLEMENTAL FILE

Additional Table A1: Thematic analyses and example ideas generated through NGT on potential benefits of increased federal access to state and local NSSP data, 2021.

Q1: In what ways can increased federal access to state syndromic surveillance data at the state or local level benefit or support state public health activities?
**1.1 - Technical assistance + expertise**
– *Even in states with dedicated staff there may not be appropriate resources to examine all potential issues on granular level*<br>– *Support states without expertise in SyS with analysis/CDC can assist*<br>– *Technical assistance in creating and standardizing syndromes for consistency across jurisdictions and support for less-resourced jurisdictions"*
**1.2 - Enhanced federal surveillance capacity (e.g., providing national pictures, completing data request normally handled by states, increased cross-jurisdictional awareness)**
– *"Easier to depict the national landscape of what's happening and trends (especially geographically) - allocation of resources, early warning*<br>– *Timely identification of novel/emerging health issues that cross jurisdictional boundaries*<br>– *In states without the ability to monitor data routinely, NSSP might be able to point out issues for state follow up that would otherwise be missed."*
**1.3 - Improved cross-jurisdiction collaboration efforts**
– *Collaborate on analysis and publications that impact more than one state, across jurisdictions, etc.*<br>– *Develop best practices and compare jurisdiction-jurisdiction collaboration/techniques*<br>– *Build powerful collaborations - tribal/local/state/federal that cross jurisdictional boundaries - border issues, tribal issues,*
**1.4 - Enhanced State Capacity**
– *More ability to train and onboard new staff in lower resource states or others without dedicated staff*<br>– *During large emergencies there is a potential benefit to having additional eyes on data*<br>– *Technical assistance in creating and standardizing syndromes for consistency across jurisdictions and support for less-resourced jurisdictions*
**1.5 - Improved Syndromic Surveillance Practice**
– *Streamlining expectations from healthcare providers and the public and what info can be shared*<br>– *If the federal partner is using one system this allows for increased interoperability in a*

way since all data is flowing or being used and analyzed through that one platform vs. many at state/local levels and then can be shared back with the participating jurisdictions comprehensive visibility into the state of syndromic trends at the state or local level,<br>– *More robust query and visualization options in NSSP ESSENCE based on the same level of access state/locals have.*

Additional Table A2: Thematic analyses and example ideas generated through NGT on potential concerns of increased federal access to state and local NSSP data, 2021.

Q2: What concerns you about increasing federal access to state syndromic surveillance data at the state or local level?
**2.1 - Increasing the burden on jurisdictions**
– *...High frequency of requests to states or locals to examine signals or other indicators of low importance or no value. (from a state perspective)*<br>– *CDC announcing something before state or locals know what is going to be made publicly available and being inundated with additional questions that you are not anticipating from the public.*<br>– *Situational awareness - double edge sword - too much info currently, can't stay on top of changing environment/situations*
**2.2 - Misinterpretation of data**
– *Inappropriate types and levels of analysis given the limitations of the data, syndrome applicability, or relationship between facilities and jurisdiction.*<br>– *Inappropriate comparisons that are seemingly at a geographic level but in reality are at a facility level because of data content, population characteristics, etc.*<br>– *CDC conducting analysis without understanding what caveats and limitations there are to the data and not talking to state/locals to be considered with analysis*
**2.3 - Publishing the data can decrease jurisdictional credibility**
– *Even beyond FOIA, CDC during COVID released large amounts of data to press, who ran analysis that then contradicted state analysis. Time spent clarifying the data and discrepancies. Costs time and credibility of state efforts. Pressure on states to report publicly and accurately likely higher than pressure on CDC.*<br>– *CDC writing, publishing, presenting on state/local the pandemic, epidemic, event, outbreak, surveillance area of interest without approval, notification, or permission.*
**2.4 - Inadequate, excessive, or inappropriate communication regarding data uses**
– *...NSSP/CDC releasing or discussing local data (meaning below a state level) with*

policymakers from those jurisdictions without state or local input/awareness.<br>– *Inadequate communication with local site administrators. The cadence and content of communications, method of communication.*<br>– *Not knowing that our data is being used and how it was being used;*
**2.5 - Negative effect on collaborations leading to presentations or publications**
– *Publications of data or analysis using state, local, or facility based data without state collaboration.*<br>– *The emphasis on collaboration across CDC programs with the community dissolves for any use of the syndromic data.*
**2.6 - Federal government independently sharing data or initiating public health action without notifying states**
– *… NSSP identifies issues and then passes it up the chain within CDC or HHS without early input from jurisdictions.*<br>– *CDC contacting our hospitals/facilities and conducting an investigation without approval, notification, or permission.*<br>– *Not knowing how CDC or any other federal partners that access to the data will use it*
**2.7 - Privacy and confidentiality concerns, including data sensitivity, restriction of certain fields, and public perception of increased data sharing**
– *…Data details is extremely sensitive. That level without restrictions (individual users for short time period, limited fields accessed) would be a hard no.*<br>– *How we account for sharing of rural locations and what protections are in place in terms of the analyses and display of that data or information*<br>– *Risk of unlimited data mining*<br>– *How will the data collected + used be utilized by CDC, other federal agencies*
**2.8 - FOIA**
– *Concern that data would have to be released due to FOIA*<br>– *FOIA and info release w/o state, local, tribal knowledge/control*
**2.9 - Adequacy of and adherence to data sharing rules (including agreements codes of conduct, etc.)**
– *Concern that any DUA agreement that could be put into place with NSSP would not apply to the centers or program areas who are providing funding.*<br>– *New CDC may not have full understanding on code of conduct (i.e., what they should and shouldn't post)*<br>– *Oversight of data linkage*

Additional Table A3: Thematic analyses and example ideas generated through NGT on potential policy options to address concerns of increased federal access to state and local NSSP data, 2021.

Q3: What rules, restrictions, guidelines, or codes of conduct could be implemented in the NSSP DUA or CDC policies that might address a concern addressed by you or a fellow workgroup member?
**3.1 - Restrict data access for specific purposes or events**
– *Access to data should be at the specific individual user level, for defined time periods, for specific purposes.*<br>– *Sharing should be for defined periods of time for specific users for specific purposes (Too open-ended)*
**3.2 - Establish audit and documentation process for data access and analysis**
– *…Code of conduct like Richard Hopkins' version-but with modifications. Needs to include process for removal of access.*<br>– *Auditing and documentation of staff access and queries of state/local data.*<br>– *Audit trail of where, how, and to whom data was shared.*
**3.3 - Restrict data access to specific users (as opposed to groups of users)**
– *Access to data should be at the specific individual user level, for defined time periods, for specific purposes.*<br>– *… States need authority for removing state level data access based on a predefined set of criteria/issues- this would exist even after access was originally granted*
**3.4 - Make DUA applicable to all federal recipients of NSSP data**
– *DUA need to include specific provisions for data re-release (if any) and that includes to internal CDC staff outside of NSSP approved staff, other federal agencies, and contractors.*<br>– *…DUA be for all of CDC and all data sources in the BioSense Platform.*<br>– *What are the limits of sharing data across federal agencies/programs/different administrations and who makes that determination*
**3.5 - Involving state and local partners in data analysis**
– *Right of first refusal by states/locals on analysis plans, protocols and publications- I am not sure exactly what I mean by this but more that states have the ability to say whether they want to complete a particular analysis or review of their own data rather than CDC just doing it and telling the state about it after- or something like that.*<br>– *Collaboration with Sites, NSSP, CDC, and the NSSP CoP, must be a part of the policy.*

Decisions on the data, system, access, use, cannot be done in a bubble.<br>– *Being clear on how the info is shared and notifying states receipts of disclosures; permission/collaborative discussion granted by the state prior to disclosures*
**3.6 - Require training on code of conduct**
– *…HIPAA like training so that staff understand code of conduct*<br>– *In addition to the code of conduct which needs to be applied to all data sources in the platform, there needs to be training for specific sites. Not every Site is the same nor is it's data contributing to the platform. Respecting and including those types of caveats. Limitations, considerations, need to be built into policy.*
**3.7 - Establish restrictions on data publication**
– *No publication of data below a national level without state/local participation offered (in the analytic stage specifically). If states prefer not to participate they then need to at least sign off on the final publication. This should include national projects that use subsets of state data that then identify the facilities or states that subset came from.*<br>– *Different diseases and conditions have different policies surrounding use and publication. There is not necessarily a one size fits all definition. Something needs to be built into the larger DUA and policy to acknowledge this.*<br>– *No publishing of data publicly*
**3.8 - Create standards for removing access**
– *… States need authority for removing state level data access based on a predefined set of criteria/issues- this would exist even after access was originally granted*
**3.9 - Create communication protocols between CDC and STLTs**
– *Perhaps some sort of decision matrix by state that includes things like geographies that are problematic, severe limitations on interpretations, etc. that CDC can refer to prior to reaching out to a state for examination of an issue*<br>– *Defined communication protocols that include methods, timelines for reply, expectations of level of effort, etc.*<br>– *In the event of a multi-state outbreak/event, how will CDC disclose info across different sites and considering the varying sites (decentralized vs centralized)*
**3.10 - Allow optional participation in greater federal access**
– *Formalize the process to request the data (opt in, not opt out)*<br>– *… Misuses of the data, where a local jurisdiction wants to opt out should be allowed.*<br>– *Develop agreements with each jurisdictions to opt-in on varying levels; make it optional for jurisdictions to participate.*

3.11 - Include procedure for DUA renewal
– *There needs to be a renewal process for the DUAs. There are many changes that happen year to year, and some are huge shifts that need to be current in the DUAs used that align with current policies in place.*
**3.12 - Clarify breach responsibility**
– *Legal authorities to collect data, who is responsible in the event of a data breach*
